# Improving the Degree-Day Model for Forecasting *Locusta migratoria manilensis* (Meyen) (Orthoptera: Acridoidea)

**DOI:** 10.1371/journal.pone.0089523

**Published:** 2014-03-05

**Authors:** Xiongbing Tu, Zhihong Li, Jie Wang, Xunbing Huang, Jiwen Yang, Chunbin Fan, Huihui Wu, Qinglei Wang, Zehua Zhang

**Affiliations:** 1 State Key Laboratory for Biology of Plant Diseases and Insect Pests, Institute of Plant Protection, Chinese Academy of Agricultural Sciences, Beijing, P. R. China; 2 Department of Entomology, College of Agronomy and Biotechnology, China Agricultural University, Beijing, P.R. China; 3 Tianjin Binhai New Area of Dagang Agricultural Service Center, Tianjin, P.R. China; 4 Cangzhou Academy of Agricultural and Forestry Sciences of Hebei, Cangzhou, P.R. China; Natural Resources Canada, Canada

## Abstract

The degree-day (DD) model is an important tool for forecasting pest phenology and voltinism. Unfortunately, the DD model is inaccurate, as is the case for the Oriental migratory locust. To improve the existing DD model for this pest, we first studied locust development in seven growth chambers, each of which simulated the complete growing-season climate of a specific region in China (Baiquan, Chengde, Tumotezuoqi, Wenan, Rongan, Qiongzhong, or Qiongshan). In these seven treatments, locusts completed 0.95, 1, 1.1, 2.2, 2.95, 3.95, and 4.95 generations, respectively. Hence, in the Baiquan (700), Rongan (2400), Qiongzhong (3200), and Qiongshan (2400) treatments, the final generation were unable to lay eggs. In a second experiment, we reared locusts for a full generation in growth chambers, at different constant temperatures. This experiment provided two important findings. First, temperatures between 32 and 42°C did not influence locust development rate. Hence, the additional heat provided by temperatures above 32°C did not add to the total heat units acquired by the insects, according to the traditional DD model. Instead, temperatures above 32°C represent overflow heat, and can not be included when calculating total heat acquired during development. We also noted that females raised at constant 21°C failed to oviposit. Hence, temperatures lower than 21°C should be deducted when calculating total heat acquired during adult development. Using our experimental findings, we next micmiked 24-h temperature curve and constructed a new DD model based on a 24-h temperature integral calculation. We then compared our new model with the traditional DD model, results showed the DD deviation was 166 heat units in Langfang during 2011. At last we recalculated the heat by our new DD model, which better predicted the results from our first growth chamber experiment.

## Introduction

Many mathematical models utilizing heat-summation are widely used in Integrated Pest Management (IPM) to forecast and predict pest insect phenology and voltinism [1]. Over the years, numerous authors have worked to improve the accuracy of such models. Simpson [2] proposed the accumulated temperature constant relationship and the inverse symmetry curve. Ludwig [3] illustrated that varying and constant temperatures influenced *Popillia japonica* development differently. Davidson [4] used a logistic curve to illustrate the relationship between growth rate and temperature, and Pradhan [5] proposed a formula index. Yang *et al.* [6] developed a weighted calculation method for variable temperatures and the natural accumulated temperature. Arnold [7] proposed a sine-curve model based on maximum and minimum temperature to estimate heat units. Schoolfield and coworkers [8] modified the Sharpe-Michele model. Wagner *et al.* [9] presented easy instructions for the use of the Sharpe-Michele model and designed a computer program to determine the correct number of parameters to be used in the model for a given data set. Recently, de Jong and van der Have [10] used the Sharpe-Michele model to assess the temperature dependence of development rate, growth rate, and size from biophysics to adaptation. All of these authors made important contributions to degree-days calculation.

Degree-day (DD) model is widely used in theoretical and basic science [11]–[14]. For example, to understand development, life history, ecology, species adaptations and biogeography, phenotypic plasticity, and physiological evolution, their widest use is applied; i.e., forecasting pest and crop phenology [10], [15]. The ability of the DD model to accurately predict pest occurrence ranges from very good to poor, depending on the specific pest and model used [16]–[18]. Hence, there is continuous effort to improve the model, which fails for any number of reasons. For example, how one calculates DD can strongly influence the accuracy of results [19]–[21], e.g., using the highest and lowest temperatures rather than the average temperature when calculating the DD. Likewise, there are numerous other factors that can alter or mitigate the influence of ambient temperature on development, including photoperiod, population density, pathogens, predators, competition, nutrition, moisture, thermoregulation, acclimation, etc [22]–[29]. In addition, the traditional DD model fails when the relationship between temperature and development rate is not linear over the viable range. For example, in *Chrysopasinica*, development rate increases non-linearly at temperatures between 30 and 32°C [30]. Thus, the upper temperature limit during insect development is important, while temperature trends during the growth season can be simulated by using the Monte Carlo method and then used as input for generating degree-day model [9], [31]–[32].

There are lots of models including Briere model, Lactin model, Logan model, Taylor model, etc., developed to study temperature dependence of development rate. Among the various models, each has advantages and disadvantages [32]–[33]. For example, they can describe development rate vary trends at different temperatures. However, in the Briere model and Lactin model, the initial values of the parameter are not set based on a reasonable explanation, while in the Logan model and Taylor model, they were unable to estimate the lower development threshold temperature [34]. Thus, with a nonlinear solution, this method obtains an approximate, rather than an exact solution [17]–[18], [35]. Usually the predictions from non-linear models are compared and validated with experimental data, as in the case of the experimental derived development upper cut off. We consider that these models will generate different outcomes when they are used to calculate the upper development threshold temperature and DD for an insect, mainly because the independent variables are set inaccurately [17]–[18], [36]–[37].

In this paper, we develop a modified DD model for the Oriental Migratory Locust, *Locusta migratoria manilensis* Meyen. This pest is widespread throughout Asia, Africa, Europe, Australia, and New Zealand, where it causes severe damage to cereal crops [38]–[43]. Occurrence and distribution records of *L. m. manilensis* can be traced back for 3000 years in China, where locust plagues are the three main natural disasters, along with floods and droughts [44]–[45]. IPM is the main strategy used to control the locust population [46], and DD-forecasting is an important component of this strategy [14].Unfortunately, current locust DD model is inaccurate, with typical discrepancies of 10 d or more between predicted vs. actual field phenology [47].For this reason, we investigated development in the Oriental migratory locust, with the goal to improve the DD model for this insect. We first reared locusts in environmental chambers which simulated the climate in various regions in China. We also reared locusts at various constant temperatures and carefully monitored there development. Based on these laboratory results, we calculated the DD for migratory locust development using the integral calculation method, and developed a new model for predicting locust development. When tested against locust voltinism, the new model was more accurate than the old model.

## Materials and Methods

### Study organism

We studied the oriental migratory locust, *Locusta migrotoria manilensis* Meyen. Eggs were collected in November (Autumn locust) 2008 from fields in near Cangzhou City (N38°30′33.46″, E117°25′32.85″), Hebei Province, China, a known breeding area for *L. m. manilensis*. Collected eggs were transferred to the Institute of Plant Protection, Chinese Academy of Agricultural Sciences, Beijing. Then, during the next year, we reared several successive generations in the laboratory as per Tu *et al*. [48]. In late 2009, we collected eggs from this laboratory colony and kept them at 4°C for three months to use in this study.

The location (N38°30′33.46″, E117°25′32.85″) which is covered with saline-alkali soil is nearby the Bohai Sea. We have got the permission for us to conduct the field studies by Cangzhou Academy of Agriculture and Forestry Sciences of Hebei province, who is the authority department responsible for pest control in agriculture and forestry land, also with the protection of wildlife in Cangzhou. With the help of Dr. Qinglei Wang (Cangzhou Academy of Agriculture and Forestry Sciences), we collected eggs in Autumn for our laboratory experiment. This location is a natural ecosystem, it is not involving endangered or protected species during the field studies.

### Using DD to predict locust voltinism

To investigate the relationship between the degree-days (DD) and voltinism (the number of generations that can be produced in a population in one year), we raised the oriental migratory locust in growth cabinets (PRX-350B-30). We used seven different cabinets, each set to a daily temperature and photoperiod cycle that mimicked the natural daily temperature and photoperiod cycles of a specific location in China: Baiquan (BQ), Chengde (CD), Tumotezuoqi (TM), Wenan (WA), Rongan (RA), Qiongzhong (QZ), and Qiongshan (QS) (Table 1). We chose these seven locations, because they encompassed most of the latitude available in China, and corresponded to seasonal DD of 700, 800, 900, 1600, 2400, 3200, and 4000 heat units, respectively, based on the previously calculated lower thermal development threshold of 14.2°C for *L. m. manilensis*. The seven locations (DD) chosen include four (BQ, RA, QZ, QS) where the oriental migratory locust is unable to breed, and three (CD, TM, WA) where the locust is able to breed. The chosen locations also included latitudes with climates that could support one (CD & TM) or more than one (WA) locust generations per year [47], [49]. Hence, we modeled latitudes that lacked natural breeding populations of oriental locust, as well as univoltine and multivoltine sites. This allowed us to test the validity of our DD model for predicting locust biogeography and voltinity. Each chamber tested a different DD, and represented a different treatment.

**Table 1:** Seven locations in China modeled in this study, and the average heat units (degree-days) available per growing season at each site, based on an estimated lower thermal threshold for development of 14.2°C for *Locusta migratoria manilensis* Meyen.

Location	BQ	CD	TM	WA	RA	QZ	QS
Longitude	E126°04′	E117°58′	E111°08′	E116°27′	E109°22′	E109°50′	E110°20′
Latitude	N47°37′	N40°57′	N40°43′	N38°52′	N24°14′	N19°03′	N19°59′
Available degree-days	698	820	902	1627	2392	3248	3898

Baiquan (BQ), Chengde (CD), Tumotezuoqi (TM), Wenan (WA), Rongan (RA), Qiongzhong (QZ), and Qiongshan (QS).

**Figure 1:**
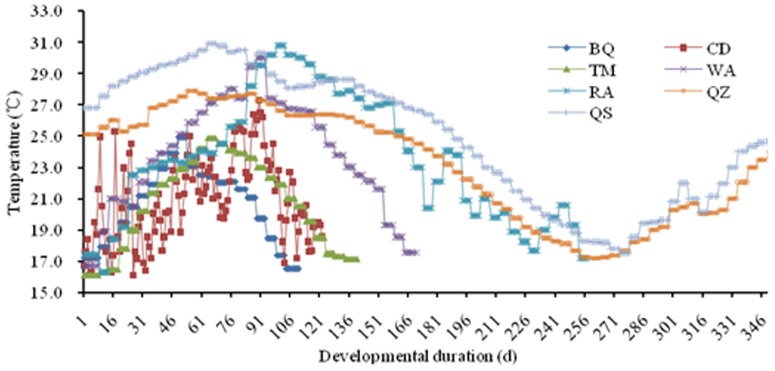
The mimicked temperatures in different growth chambers. Seven locations in China modeled in this study, Baiquan (BQ), Chengde (CD), Tumotezuoqi (TM), Wenan (WA), Rongan (RA), Qiongzhong (QZ), and Qiongshan (QS). Mean temperatures were obtained from (http://cdc.cma.gov.cn/home.do) throughout the growing season for each location based on an estimated lower thermal threshold for development of 14.2°C for *Locusta migratoria manilensis* Meyen. Growth chamber temperatures in Chengde changed daily while in other locations changed every 10 d.To simulate variable environmental temperatures, we designed a variable range of ‘±5°C’ for the daily 24-h temperature change.

In the field, temperature and photoperiod vary throughout the season, and temperature cycles on a 24-h basis. Using recorded weather data (http://cdc.cma.gov.cn/home.do), we estimated mean daily temperatures and photoperiods throughout the growing season for each location (Fig. 1). We then set each of our growth cabinets to mimic the hourly and seasonal conditions that occur in the field during the locust growing season. Hence, growth chamber temperatures changed hourly and photoperiod changed every 10 d throughout the experiment (Table 2). The relative humidity (RH) was kept at ∼60% for eggs and ∼80% for nymph and adults. Note that each chamber (each treatment) ran for a different number of days (Table 2), which matched the local locust growing season (i.e., number of days in the field at that location where the mean daily temperature exceeded 14.2°C). Hence, the growing season for *L. m.manilensis* at high latitude BQ is only about 110 d, whereas low latitude QS provides a 360-d growing season. Therefore, the growing chamber that simulated the BQ climate ran for only 110 d, whereas the QS treatment ran for a year (Table 2).

**Table 2:** Photoperiod regimes used in the experiment.

Day (d)	BQ	CD	TM	WA	RA	QZ	QS
1–10	14∶10	13.5∶10.5	13.5∶10.5	13.5∶10.5	13∶11	12.5∶11.5	12.5∶11.5
11–20	14∶10	13.5∶10.5	13.5∶10.5	13.5∶10.5	13∶11	12.5∶11.5	12.5∶11.5
21–30	14∶10	14∶10	13.5∶10.5	13.5∶10.5	13∶11	12.5∶11.5	12.5∶11.5
31–40	14∶10	14∶10	13.5∶10.5	13.5∶10.5	13∶11	12.5∶11.5	12.5∶11.5
41–50	14∶10	14∶10	13.5∶10.5	13.5∶10.5	13∶11	12.5∶11.5	12.5∶11.5
51–60	15∶9	14.5∶9.5	14.5∶9.5	14.5∶9.5	13.5∶10.5	13∶11	13∶11
61–70	15∶9	14.5∶9.5	14.5∶9.5	14.5∶9.5	13.5∶10.5	13∶11	13∶11
71–80	15∶9	15∶9	14.5∶9.5	14.5∶9.5	13.5∶10.5	13∶11	13∶11
81–90	16∶8	15∶9	14.5∶9.5	14.5∶9.5	13.5∶10.5	13∶11	13∶11
90–100	16∶8	15∶9	14.5∶9.5	14.5∶9.5	13.5∶10.5	13∶11	13∶11
101–110	16∶8	15∶9	14.5∶9.5	14.5∶9.5	13.5∶10.5	13∶11	13∶11
111–120		15∶9	14.5∶9.5	14.5∶9.5	13.5∶10.5	13∶11	13∶11
121–130		15∶9	14.5∶9.5	14.5∶9.5	13.5∶10.5	13∶11	13∶11
131–140			14.5∶9.5	14∶10	13.5∶10.5	13∶11	13∶11
141–150				14∶10	13.5∶10.5	13∶11	13∶11
151–160				14∶10	13.5∶10.5	13∶11	13∶11
161–170				14∶10	13.5∶10.5	13∶11	13∶11
171–180					13∶11	12.5∶11.5	12.5∶11.5
181–190					13∶11	12.5∶11.5	12.5∶11.5
191–200					13∶11	12.5∶11.5	12.5∶11.5
201–210					12∶12	12∶12	12∶12
211–220					12∶12	12∶12	12∶12
221–230					12∶12	12∶12	12∶12
231–240					11∶13	12∶12	12∶12
241–250					11∶13	12∶12	12∶12
251–260					11∶13	12∶12	12∶12
261–270						11∶13	11∶13
271–280						11∶13	11∶13
281–290						11∶13	11∶13
291–300						11∶13	11∶13
301–310						11∶13	11∶13
311–320						11∶13	11∶13
321–330						10∶14	10∶14
331–340						10∶14	10∶14
341–350						10∶14	10∶14
351–360						13∶11	13∶11

Each growth chamber was set to simulate the natural seasonal photoperiod pattern of a different location in China: Baiquan (BQ), Chengde (CD), Tumotezuoqi (TM), Wenan (WA), Rongan (RA), Qiongzhong (QZ), or Qiongshan (QS). The first column (Day) divides the experimental period into successive 10-d segments. The other columns show the photoperiod settings for each growth chamber during each 10-d period. Note that each location gives photoperiods for only those weeks in the field when air temperature exceeded 14.2°C. Photoperiod data derived from (http://cdc.cma.gov.cn/home.do).

To start the experiment, we transferred about 300 locust eggs into each of the seven growth cabinets. The resulting nymphs and adults were kept in 50×50×60-cm tall cages and fed twice each day with freshly cut wheat leaves (*Triticum sativa* L.) and once each day with artificial diet (100 g wheat bran+5 ml corn oil+vitamins B and C) [48]. The bottom of each cage contained a 20-cm diameter round hole, under which was placed a 20-cm diameter ×9-cm tall container with compacted sand (3.5 kg sterile sand+0.6 L sterile water) which allowed females to oviposit ad lib. The container was replaced twice a day to ensure space for eggs-laying. We carefully recorded the duration, mortality of each stage, and observerd voltinisms of each treatment during the experiment. Generation times were calculateded by weighted average method [14].

### Effects of temperature on locust development

To examine the effects of temperature (especially higher temperature) on locust development, locust eggs (30 eggs/duplicate treatment, five duplicates/treatment) were transferred to growth cabinets, which were maintained at constant temperatures of (18, 21, 24, 27, 29, 30, 31, 32, 34, 36, 38, 40, 41, or 42)±0.5°C, with 60±5% RH. We recorded the development durations and the survival rates of eggs, nymphs and adults in each treatment. The nymphs and adults were reared as described at (18, 21, 24, 27, 29, 30, 31, 32, 34, 36, 38, 40, 41, or 42)±0.5°C, with 80±5% RH and a 12∶12 L∶D photoperiod. The lower threshold temperature (LTT) for oocytes development was estimated.

### Effects of temperature on adult egg-laying

We tested the effects of temperature on adult egg-laying. Locust eggs were transferred to growth cabinets which were maintained at seven constant temperatures (18, 21, 22, 23, 24, 27, and 30°C). Ten freshly molted adults (5 ♂, 5 ♀) were obtained from nymphs reared at each of the seven temperatures and maintained in incubators at the same constant temperature and conditions as before. The adults were confined in pairs (1 ♂+1 ♀) in clear 500 ml plastic containers (five pairs per temperature). For each female adult, we recorded the intervals from adult molt to the first oviposition, percentage of females that laid eggs, number of egg-pods laid and adult longevity. The containers lacked sand, so the females laid their egg pods on the floor or the sides of the containers.

### DD integral calculation and DD model improving

We recorded 24-h temperature data on each day using a HOBO Pro v2 logger, where the stability was <0.1°C per year. This instrument also had sufficient memory to record over 42,000 12-bit measurements. Low threshold temperatures for different development stage (i) were defined as ‘*C_i_*’, so only temperature higher than ‘*C_i_*’ was considered for analysis and heat accumulation. First, we modified the 24-h temperature change function (f_t_) using Matlab R2011b. The program script was as follows:

t = 1∶24; %(‘t’ as daily 24 h, 1≤t≤24)

d = [data]; %(‘d’ as daily 24 h temperature data)

p_n_ = polyfit(t,d,n); % (‘n’ as the power of the function, generally 1≤ n ≤6)

poly2str(p_n_,‘t’) %(obtains the function ‘f_t_’)

Second, we calculated the area of the temperature higher than ‘*C_i_*’ in the figure, as follows:

solve (‘f_t_ = *C_i_*’) %obtains the intersect of ‘a’ and ‘b’ between y = *C_i_* and f_t_

syms t;

int (‘f_t_’, t, a, b) %get the area S_1_ from ‘a’ to ‘b’, S_1_ = ∫T(a,b), ‘T’ is the temperature at time ‘t’

S = [S_1_- *C_i_* *(b-a)]/24%‘S’ is the required DD

In this method, we used degree-hours instead of the traditional degree-days to calculate heat units, as follows: S = ∑(T- *C_i_*)/24.

Using our experimental findings, we could get some sensitive characteristic parameters of migratory locust (i.e., the upper threshold temperature and prevent egg-laying temperature). These parameters and integral calculation were conducted and used to improve the DD model.

### Different DD calculating methods comparision

We compared the accuracy of four different DD calculating methods based on either: (1) the daily mean temperature, (2) max-min temperature [50], (3) 24-hours mean temperature, or (4) integral calculation of 24-hours temperature, based on the previously calculated lower thermal development threshold of 14.2°C for *L. m. manilensis* [51]. Then we analyzed which method could simulate actual temperature variation trend.

### Standard heat units calculation and validation based on the Improved DD model

We tested the validity of our calculated DD in the field by recording environmental temperatures and life history of *L. m. manilensis* in Langfang, China in 2011. Because locust eggs survive below ground and locust nymphs and adults live above ground, we used ground temperatures for eggs and air temperatures for nymphs and adults. DD were calculated based on our improved DD model.

We further evaluated the validity of the improved DD model, by recalculating the DD for the oriental migratory locust development at each of our seven focus sites. In this analysis, we used only air temperature, because this was the basis of our growth cabinet studies.

## Results and Discussion

### Voltinism under different DD

When the oriental migratory locusts were reared in seven different growth chambers providing either 700, 800, 900, 1600, 2400, 3200, or 4000 DD, the number of complete generations that were produced varied dramatically among the different treatments (Table 3). For example, locusts were unable to complete a full generation when reared in the chamber that provided only 700 DD, and which simulated the climate of Baiquan, China (BQ). In contrast, locust completed 1.1 generations in the 900 DD treatment, which mimicked daily temperatures from cool, high latitude Tumotezuoqi (TM). Likewise, locusts reared in the 4000 DD growth chamber, which mimicked daily temperatures from warm, low latitude Qiongshan (QS), completed 4.95 generations (Table 3). Thus, in this laboratory experiment, the number of heat units (DD) available strongly influenced the number of locust generations produced. But there was substantial individual variation in generations completed within treatments. For example, only low proportion eggs laid by the generation I females could keep on developing and reach to the 1^st^ instar nymphs in the cool 900 DD treatment, whereas those reared in the warm 3200 DD growth chamber all locusts of the generation IV reached adult stage and lived as adults for a long time (∼156 days), but without laying a single egg-pod. So “0.95” indicates the locusts were not able to complete full generation under those temperature and photoperiod conditions, “0.1” and “0.2” indicate only little of eggs hatched while most of them could stay in egg stage and overwintered. In such a case, the proportion of development reached is given. Hence, for the BQ treatment, locusts developed only 95% ( = 0.95) of the way to a full generation. In contrast, the TM growth chamber produced 1.1 generations (Table 3).

**Table 3:** Voltinism of L.m.manilensis reared in the laboratory under seven different simulated “climates”, each representing a different location in China: Baiquan (BQ), Chengde (CD), Tumotezuoqi (TM), Wenan (WA), Rongan (RA), Qiongzhong (QZ), or Qiongshan (QS).

Location and DD modeled	Theoretical generations by traditional DD model	Generations completed in laboratory	Generations Completed in field
BQ (700)	0	0.95 (egg-to-adult)	No locust distribution
CD (800)	1	1 (one egg-to-egg generation)	1
TM(900)	1.1	1.1 (one egg-to-egg generation + egg-to-1^st^ instar nymph of the II generation)	1
WA (1600)	2	2.2 (two egg-to-egg generations + egg-to-2nd instar nymph of the III generation)	2
RA (2400)	3	2.95 (two egg-to-egg generations + egg-to-adult of the III generation)	No locust distribution
QZ (3200)	4	3.95 (three egg-to-egg generations + egg-to-adult of the IV generation)	No locust distribution
QS (4000)	5	4.95 (four egg-to-egg generations + egg-to-adult of the V generation)	No locust distribution

I, II, III, IV, and V refers to the 1st, 2nd, 3rd, 4th, and 5th generation, respectively.

Developmental asynchrony within a single generation produced substantial overlap between generations. For example, for locust reared in the 1600-DD Wenan simulation, the overlap period between the 1^st^ and 2^nd^ generation was about 17d. As such, some 2^nd^ generation hatchings were already 17-d old by the time that the slowest 1^st^ generation female laid her 1^st^ egg pod.

### Mismatch between predicted voltinism and realized voltinism

Our laboratory experiment testing seven different temperature treatments found substantial differences between the predicted number of generations and the realized number of generations (Table 3). Previous work estimated that 800 DD above a critical low-temperature threshold of 14.2°C were necessary for the Oriental locust to undergo one complete generation [51]. Hence, the 800, 900, 1600, 2400, 3200, and 4000-DD treatments (Table 3) should have produced 1, 1.1, 2, 3, 4, and 5 generations, respectively. However they did not (Table 3). For example, the QZ treatment provided 3200 DD, which should have produced at least four complete generations, but instead produced only three egg-to-egg generations and the 4^th^ unaccomplished generation. Thus there would be: (1) Mismatch between predicted and realized voltinism according to the traditional DD model. (2) Some characteristics of migratory locust response to temperature change were unknown. We therefore attempted to identify and correct these sources of errors, and improve the accuracy of our predictions.

There was a substantial similarity in generations completed between laboratory simulation and field studies (Table 3), for example, four locations (BQ, RA, QZ, QS) where oriental locust is unable to breed, and three locations (CD, TM, WA) where the locust is able to breed, including the climates could support one in (CD & TM) or two in (WA) locust generations per year [47], [49]. However, overwinter eggs will enter diapause without hatching in the same year undergo the climates of (CD & TM) in field, hatchlings emergence mainly because of we have mimicked air temperature in 900 DD (TM) treatment, while the ground temperature would be suitable for them to stay in egg stage. In contrast, climate in Tianjin where could support two locust generations produced parts of hatchling emergence in October in recent years. Thus, temperature increasing in Autumn would enhance overwinter egg-hatching before entering diapause, and decrease population in the next Spring.

### Overflow temperature for locust development

**Figure 2:**
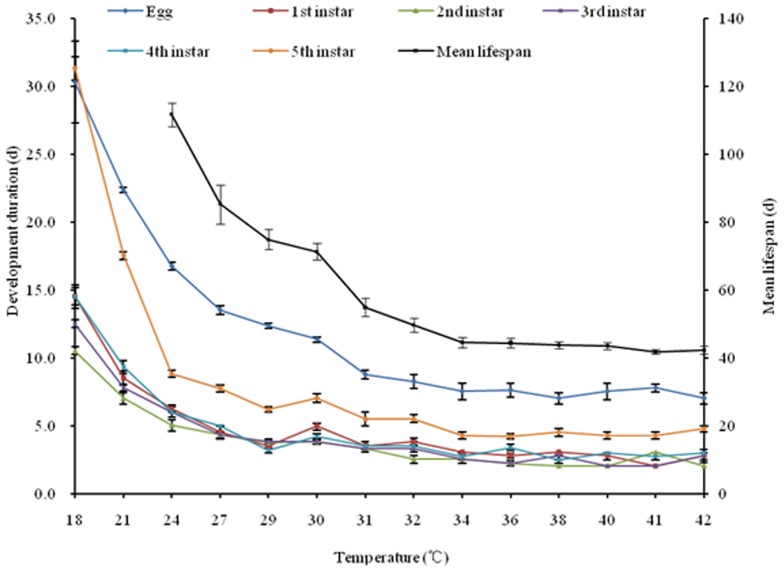
Developmental duration of locust eggs and nymphs at different constant temperatures. Each line represents the mean developmental duration of the same developmental stage at different constant temperatures.

To obtain a better understanding of the relationship between temperature and development, we studied locust development rates in growth chambers under 14 different constant temperatures ranging from 18 to 42°C (Fig. 2). Results showed that temperature strongly influenced development rates for both eggs and nymphs (Fig. 2). For example, development was significantly shorter at 32 to 42°C than at 18 to 31°C (F = 65.38, *P<*0.0001). From 18 to 32°C, development rate was a linear function of temperature (Fig. 2). In contrast, temperatures above 32°C had little effect on development rate (Fig. 2).Thus, 32°C appears to be an important inflection point for oriental locusts: above this value, higher temperatures do not produce faster development which should be defined as overflow temperature for migratory locust development. This is critical for calculating DD and estimating total effective temperatures.

**Figure 3:**
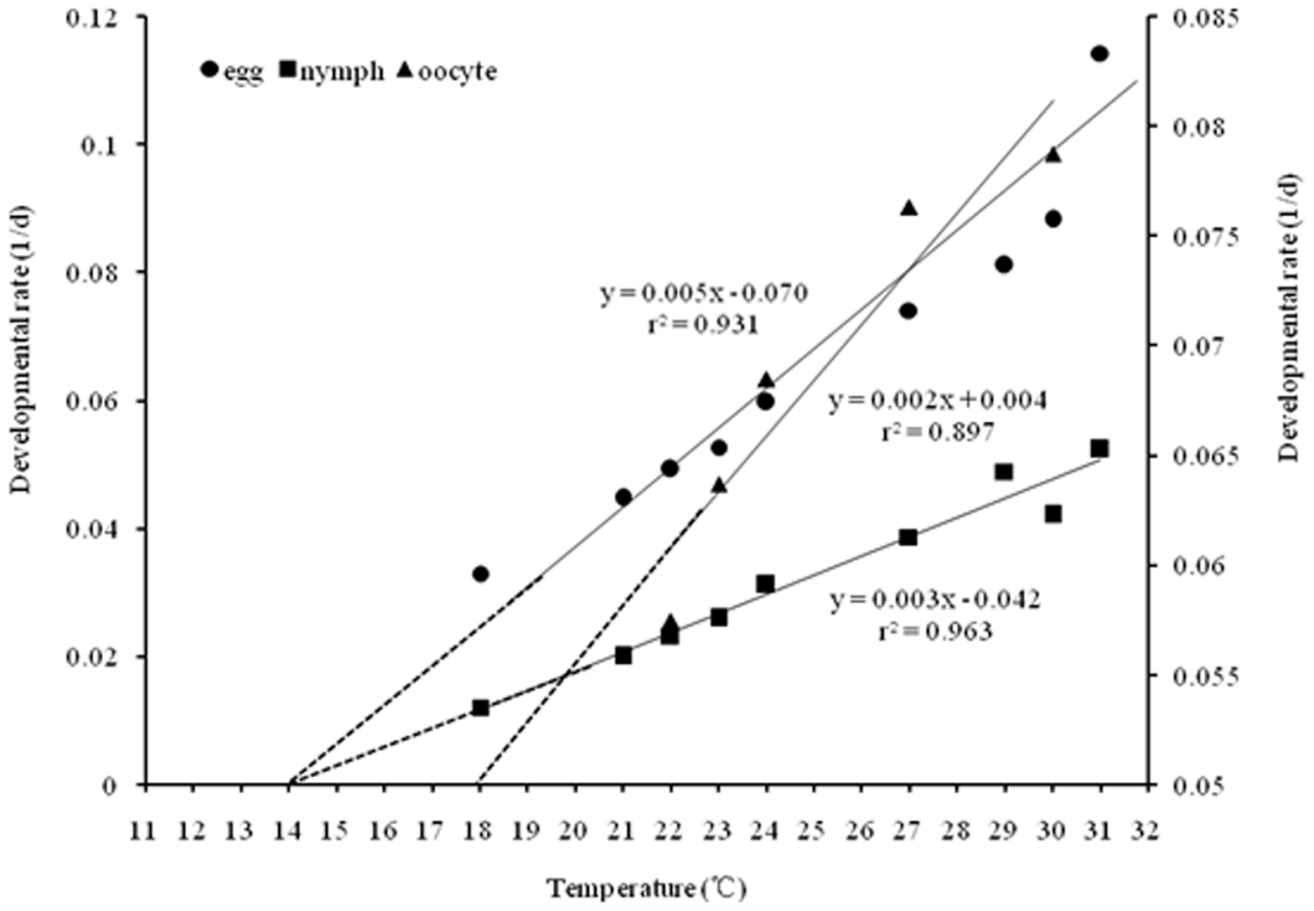
Relationship between temperature and locust (eggs, nymphs, and oocytes) developmental rate. Oocytes developmental rate based on calculating the pre-ovipositing period at 22, 23, 24, 27, and 30°C, while dashed lines show theoretical extension of regression lines to x-axis. The points where the lines intersect the x-axis represent the theoretical low temperature threshold (LTT) for development egg, and nymph was ∼14°C, while for oocyte was ∼18°C.

Eggs and nymphs developed faster when reared at higher temperatures till temperature at 32°C (Fig. 2), and this was true for eggs, nymphs and temperatures tested (Fig. 3). Development rate was a linear function of temperature from 18 to 31°C (Fig. 3), the relationship between development rate of egg (V) and temperature (T) was: V = 0.005T - 0.070, r^2^ = 0.931, while the function for nymphs (including 1^st^ to 5^th^ instar nymphs) was: V = 0.003T - 0.042, r^2^ = 0.963. Extending these development regression lines to the x-axis gives us the theoretical low temperature threshold (LTT) for development. Note that these values converge to ∼14°C for the tested eggs and nymphs (Fig. 3).

Many studies have explored the temperature relationships of various subspecies of the migratory locust, *Locusta migratoria* [40]–[41], [47], [52]–[53]. In general, the biochemical reactions are sensitive to temperature, increasing in rate of locusts with increaseing temperature [48], [54]. In the present study, the developmenta rate of locusts increased with the temperature arising from 18 to 31°C (Fig. 3). This conclusion agreed with the results calculated using the traditional DD model [14]. But the overflow temperature was never reported in previous studies [47] and it could induce predicting deviation based on the traditional DD model [14].

Lower threshold temperature (LTT) may differ considerable between immature and mature stages [28], [55], so when we predict locust development progress in field studies, the LTT of eggs, nymphs, and adults would be necessary. In the present study, we get the LTT of eggs and nymphs (Fig. 3) seems similar to the previous work as per Tu [51] which was ∼14.2°C, to ensure contextual consistence and avoid confusion, we used the LTT of 14.2°C for eggs, nymphs in this paper.

### Minimum temperature for adult egg-laying

To further investigate why the traditional DD model failed to accurately predict locust voltinism in our growth chamber experiments (Table 3), we examined the relationship between egg laying and temperature. The results (Table 4) showed that adults failed to oviposit at 18 and 21°C, there are two hypotheses: (1) The low temperature threshold (LTT) for oocyte development is above 21°C. (2) The females developed mature oocytes, but were unable to mate or oviposite, because mating or oviposition requires neural and muscle action, and 21°C is too low—i.e, the LTT for mating or oviposition (pushing eggs out of the body) muscles and nerves signals is above 21°C [28]. To investigate which hypothesis will be plausible, we examined the relationship between oocyte development and temperature (Fig. 3), result showed the LTT for oocyte development was ∼18°C (Fig. 3). As locusts can reach to adult and stay in this stage for a long time, the DD would be enough for oocyte development at 18 and 21°C, so the first hypothesis seems implausible. Thus, females require temperature higher than 21°C for reproduction behavior (mating or ovipositing) at constant temperatures.

**Table 4:** Oviposition behaviors of migratory locusts at different constant, life time temperatures, given as means ± S.E.

Temperature(°C)	Pre-ovipositing period(d)	Percentage of females that laid eggs (%)	Number of egg pods per female	Adult longevity (d)
18	——	0	0^cB^	75.0±7.2
21	——	0	0^cB^	93.0±2.9
22	17.4±0.3	80	0.8±0.2^cB^	69.8±5.5
23	15.7±0.4	100	2.0±0.3^bcB^	65.8±3.3
24	14.6±1.5	100	7.0±1.2^abAB^	62.8±2.1
27	13.1±0.4	100	10.5±2.3^aA^	45.8±5.4
30	12.7±0.3	100	12.3±2.7^aA^	36.2±2.8

See Methods section for further explanation.

Within each column, the lowercase letters indicate significant differences at *P*<0.05, and capital letters indicate significant differences at *P*<0.01. Note that at 30°C, females laid their 1^st^ egg-pod about 13 d after the adult molt, whereas the 21°C females lived an average of 93 d as adults without laying a single pod.

For the treatments that produced oviposition (22, 23, 24, 27, and 30°C), the pre-oviposition intervals of female adults were 17.4, 15.7, 14.6, 13.1, and 12.7 d, respectively. While the percentages of females that laid eggs were 0, 0, 80%, 100%, 100%, 100%, and 100%, and numbers of egg-pods per female were 0, 0, 0.8, 2.0, 7.0, 10.5, and 12.3 at 18, 21, 22, 23, 24, 27, and 30°C, respectively. The difference analysis showed the minimum temperature for adult reproduction behavior should be at a point between 21 and 22°C (Table 4), but there was no difference between them (F = 8.88, *P* = 0.0002). Thus, 21°C appears to be an important inflection point for the oriental migratory locusts: under this value, lower temperatures do not produce egg-pods which should be defined as ineffective temperature for adult reproduction behavior. This is critical for calculating DD and estimating the distribution locations for the oriental migratory locusts [14].

### DD based on 24-h temperature integral calculation

**Figure 4:**
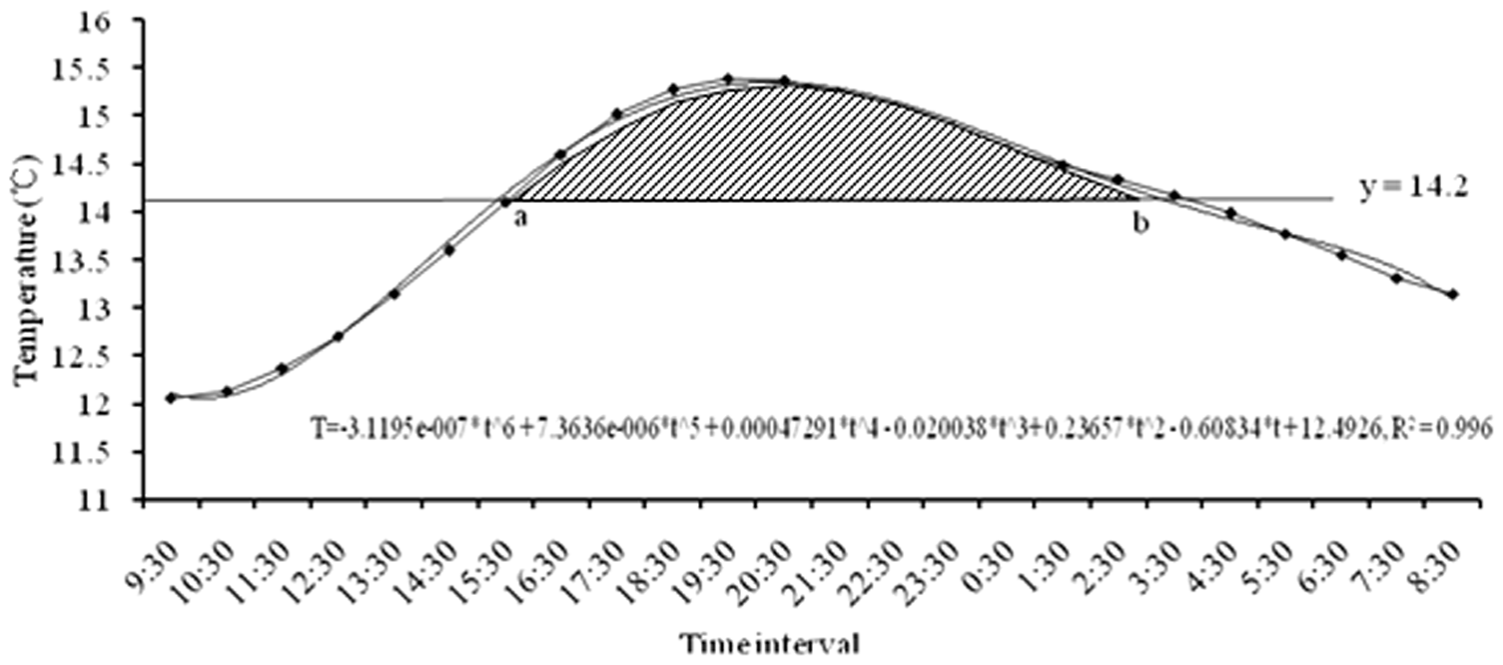
DD for locusts on April 12, 2011, in Langfang based on the integral calculation method. 24-hours ground temperature was recorded by HOBO Pro v2 logger which were used to model temperature change curve by Matlab R2011b. ‘a’ & ‘b’ represent the intersect points between y = 14.2 and the curve. The shade area was the DD for locust development in this day.

On April 12, 2011, the 24-h temperature at 5 cm underground in Langfang was as shown in Fig. 4. Using Matlab to simulate the temperature change function: f_t_ = −3.1195e−007* t∧6+7.3636e-006*t∧5+0.00047291*t∧4−0.020038*t∧3+0.23657*t∧2−0.60834*t+12.4926, R^2^ = 0.996. The results showed that a = 7.0745, b = 18.3312, and the heat unit was 0.3 DD. The function used by the integral method was: K = ∑N[∫T(a,b)−14.2*(b−a)]/24, where K is the total effective temperatures, N is the development duration, and [∫T(a,b)−14.2*(b−a)] is the area of the shaded part in Fig. 4.

**Figure 5:**
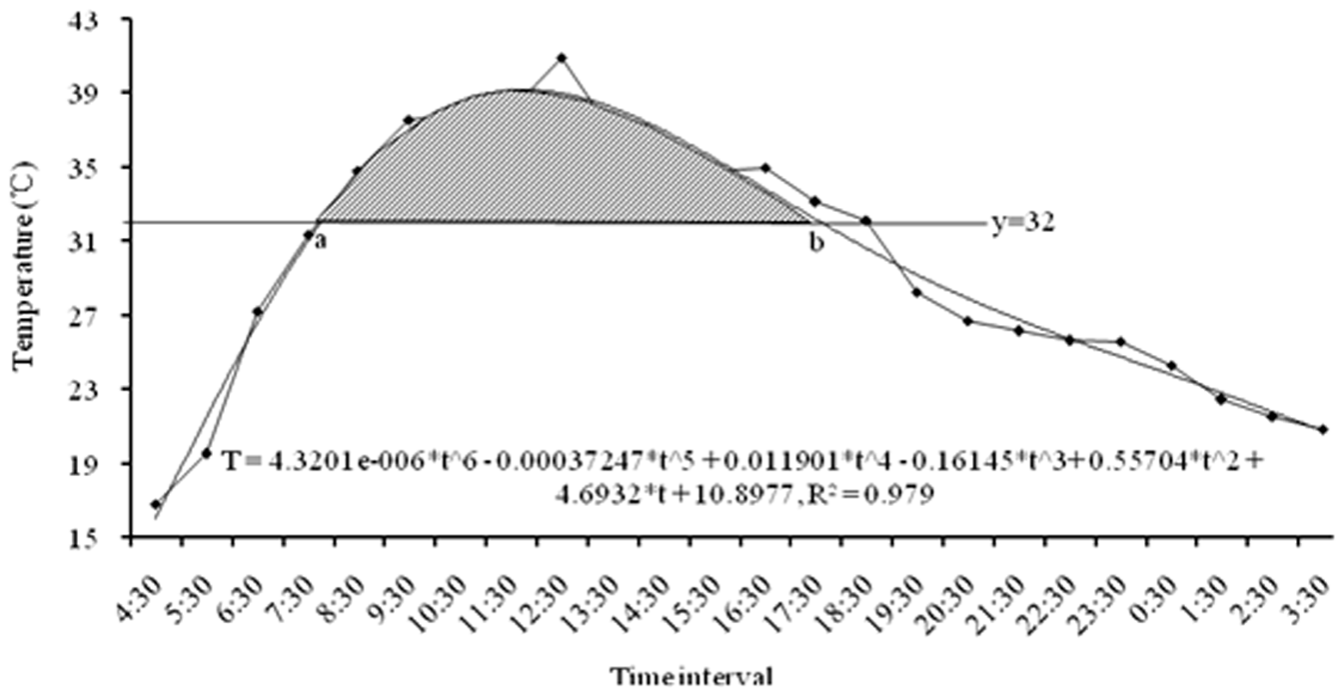
Overflow DD for locusts on July 9, 2011, in Langfang based on the integral calculation method. 24-hours air temperature was recorded by HOBO Pro v2 logger which were used to model temperature change curve by Matlab R2011b. ‘a’ & ‘b’ represent the intersect points between y = 32 and the curve. The shade area was the overflow DD for locust development in this day.

Temperatures higher than 32°C did not accelerate the development rate of the migratory locust, should not be included in the DD calculation (Fig. 5). On July 9, 2011, the 24-h temperature change is shown in Fig. 5 and the overflow temperature was 1.9 DD: a = 4.2758, b = 14.0974. The function used to calculate the overflow DD was: 
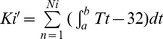
, where *i* is the development stage, *Ki'* is the overflow DD at ‘*i*’ stage, *Tt* is the temperature at time ‘*t*’ (1≤*t*≤24), ‘*a*’ and ‘*b*’ are time when the temperature is higher than 32°C during the 24-h period, and *Ni* is the development duration of ‘*i*’ stage. We have calculated the overflow DD was 38 heat units during 2011 in Langfang using this model, whereas there was no overflow DD based on the daily mean temperature. The overflow DD is also part of the invalid heat unit that causes the DD to increase nonlinearly with generations, so it has an important effect on locust development in the southern population.

### Different DD calculating methods comparision

**Figure 6:**
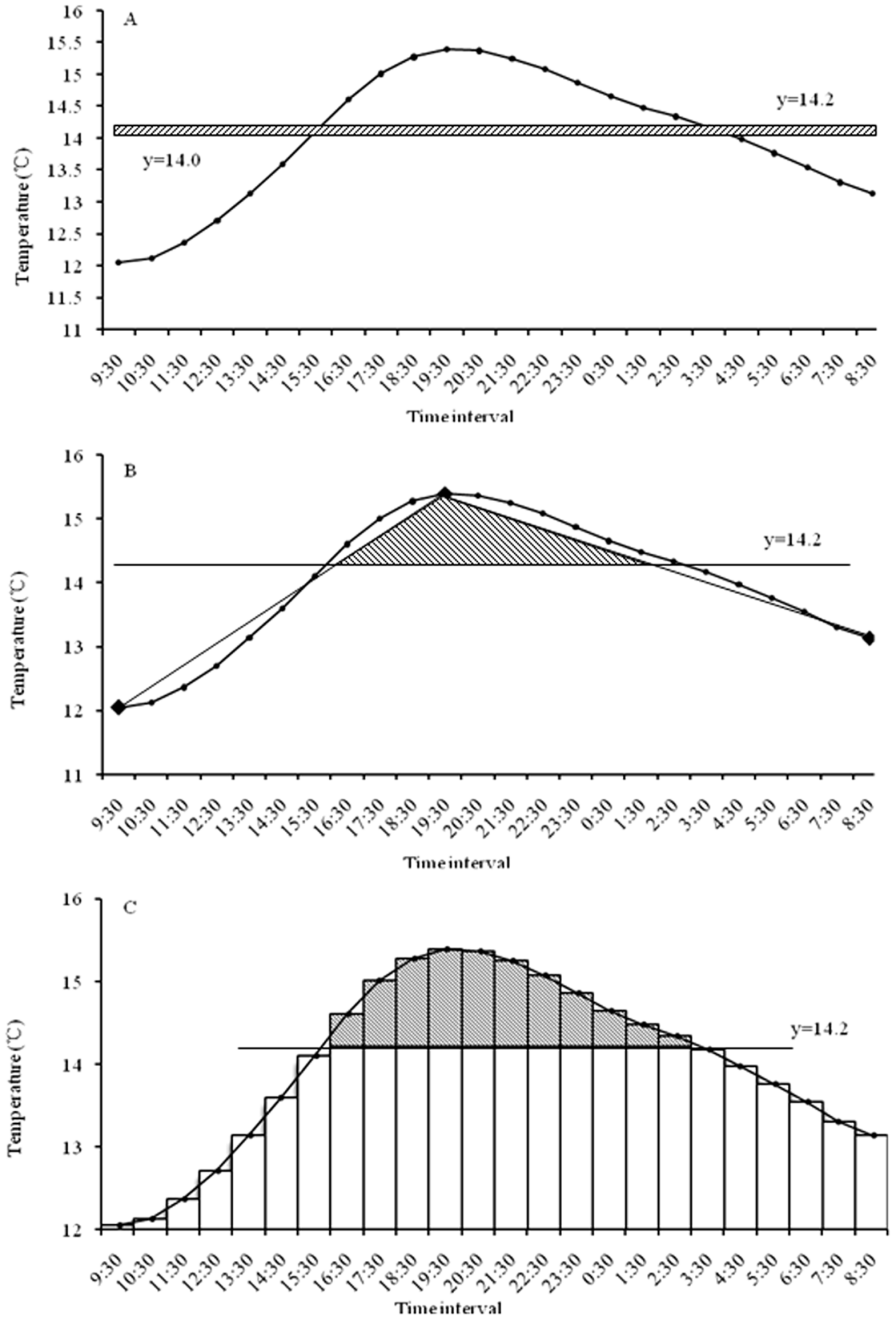
DD calculation based on mean, max-min, 24-hours temperature data. 24-hours temperature data was shown in Fig. 4. Minimum, mean and maximum temperature of this day was about 12.1, 14.0 and 15.4°C. *(A)* DD calculating based on daily mean temperature. *(B)* DD calculating based on maximum-minimum temperature. *(C)* DD calculating based on 24-hours temperature, it should be calculated as: ∑(T-14.2)/24, not the same as 24-hours integral calculation in Fig. 4.

To further investigate the accurancy of 24-hours temperature integral calculation, we compared DD calculation by four different methods. Results showed it was −0.2, 0.2, 0.3 and 0.3 DD based on daily mean temperature (Fig. 6), max-min temperature (Fig. 6), 24-hours temperature (Fig. 6), and 24-hours temperature integral calculation (Fig. 4), respectively. Two criterias were used to assess which method would be more suitable (Table 5). Results showed that 24-hours integral calculation would get the accurate time interval about temperature higher than the lower development threshold temperature and simulate the actual temperature variation trends (Fig. 4, Fig. 6). By taking 24-hours integral calculation as a contrast, the relative error of daily mean, max-min, 24-hours temperature was 167%, 33%, 0% respectively (Table 5).

**Table 5:** Comparision the different DD calculating methods based on the daily mean, max-min, 24-hours temperature, and 24-hours temperature integral calculation, whether it could describe the accurate time interval about temperature higher than the lower development threshold temperature (C) and simulate actual temperature variation trends accurately.

Calculating methods	Simulating actual temperature variation trends accurately	Describe time interval about temperature higher than C accurately	Relative error (%)
Daily mean	−	−	167
Max-min	−	−	33
24-hours temperature	−	+	0
24-hours integral calculation	+	+	0

Note ‘−’ represents ‘not’ while ‘+’ represents ‘yes’. Relative error represents the results (X) by different methods comparied to 24-hours integral calculation (Y), as follow: Relative error (%) = |X-Y|/Y*100%.

The integral calculation based on 24-hours temperature was used to calculate the insect development rate considered the effects of low temperatures in the spring and high temperatures in the summer (Fig. 4, 5), which also could describe the temperature vary trend compared to other data (Fig. 4, Fig. 6; Table 5) and get the more accurately results [20]–[21], [32], [50]. This method has solved the problem of how to set the initial values as we have 24 temperature data each day, it has eliminated the error by the Briere model, Lactin model, and several other popular models [6], [32] and got an exact solution [17]–[18], [35]. We have conducted the concept of degree-hours to calculate DD, which is more precise and available in field conditions. In theory, using degree-half an hours or degree-minutes to calcualte DD would be more accurancy. Unfortunately, they need cumbersome sampling processes. Then it is better for us to take degree-hours in field studies, because it has simplified the overall calculation process and enhanced the precision of 0.1 DD when compared with the method of Zan *et al.* [32]. Thus, the integral method could have broad applications in insect forecasting and predicting.

### Improvement of the DD model

The overflow temperature did not accelerate the development rate of locusts (Fig. 3) and temperature lower than 21°C was invalid for females reproduction behavior (Table 4), so we modified the DD model for migratory locusts in different stage as: 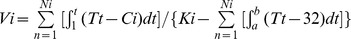, where *V_i_* is the development rate for one day at stage ‘*i*’, *Ci* is the development threshold temperature for migratory locusts at stage ‘*i*’ (for eggs and nymphs, *Ci* = 14.2°C; for adults, *Ci* = 21°C), and *K_i_* is the total effective temperatures at stage‘*i*’ or thermal constant.

Empirical DD values are not absolutely constant, even if the feeding environment and all other environmental influences are the same. Environmental influences other than temperature, such as food, population density, and all specifics, influence the number of DD, and, in addition, development rates sometimes deviate slightly from linearity. However, the number of DD to reach maturity is constant to be of biological interest. It implies that the linearity of development rate as a function of temperature is more than a statistical first approximation: it seems a biological property. Therefore, it is a biological question how linearity of development rate is caused [10]. So we proposed a standard DD calculating method based on studied the biological property of the oriental migratory locust. In the present study, invalid DD (i.e., overflow DD ineffective DD for egg-laying) should not be considered when applying the DD model to the oriental migratory locust.

### Standard heat units calculation based on the improved DD model

**Figure 7:**
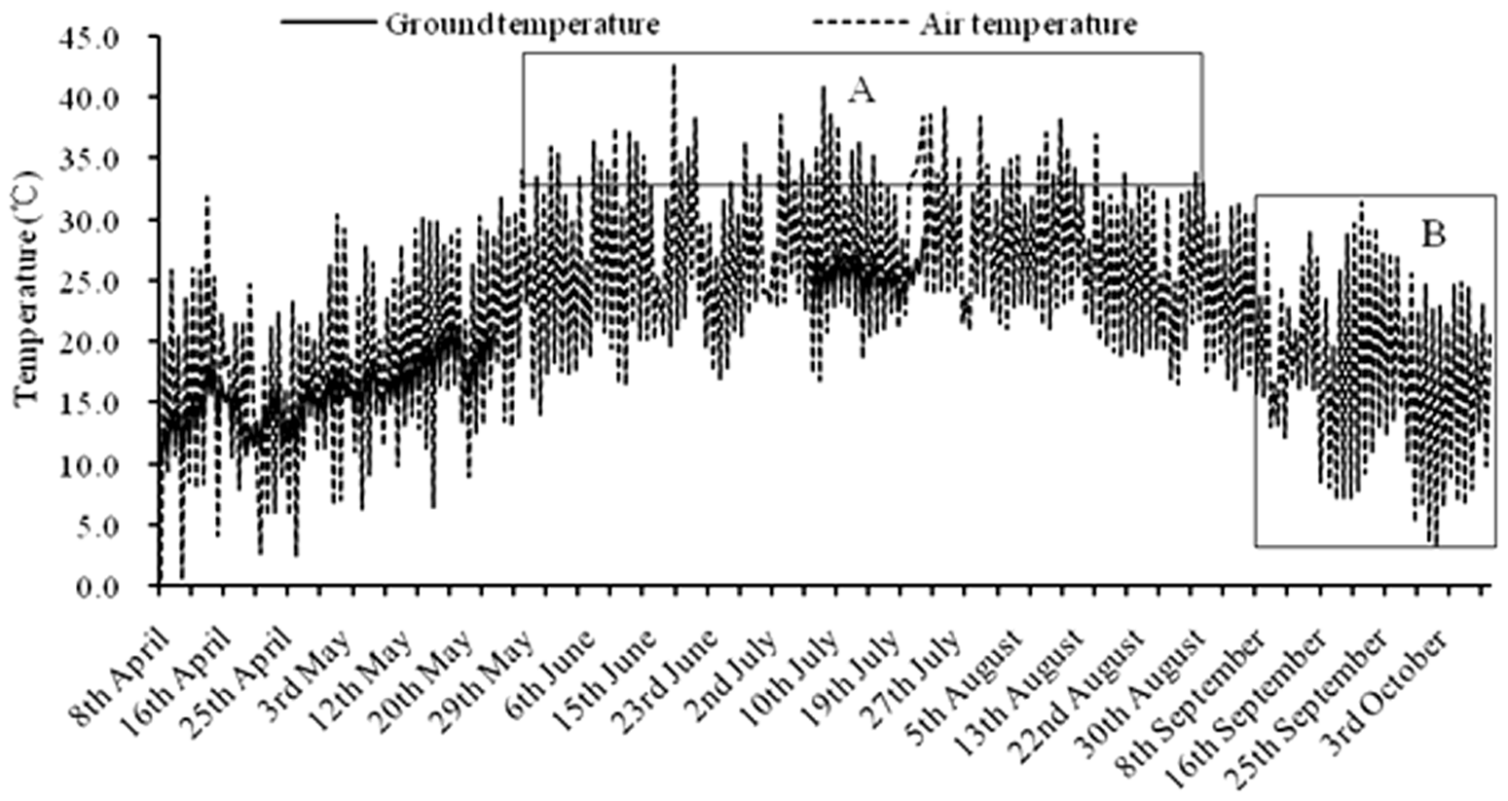
Standard DD calculating for locust accomplishing full generations based on life history of *L. m. manilensis* and temperature changes in Langfang, 2011. In field, we predicted hatchlings emergence mostly by air temperature as we lacked ground temperature data, so the DD was 1566 DD when only used air temperature (the dashed line). Instead, when we used ground temperature (the solid line) to calcualte DD for eggs, the total DD for migratory locust was 1437 heat units based on 24-hours integral calculation. *(A)* Overflow DD was 38 heat units from 28^th^ May to 5^th^ September. *(B)* Useless DD for females egg-laying was 128 heat units from 8^th^ September to 10^th^ October (these days were ovipositing periods for females, but temperature lower than 21°C).

The improved DD model showed that invalid DD should be deducted, so standard DD calculation for locust development should deduct two parts, one was to take off the overflow DD during the whole generation, while the other one was the ineffective DD when mean daily temperature was lower than 21°C for egg-laying, as depicted in Fig. 7. We analyzed the life history of *L. m. manilensis* and temperature changes in Langfang, 2011, which can support two locust generations in field [47]. Results showed that the overflow DD and ineffective DD for egg-laying was 38 and 128 heat units, respectively (Fig. 7). In other words, the DD error in this location was 166 ( = 38 +128) heat units between the improved and traditional DD model.

When ground temperature and air temperature were seperated to calculate different development stage of migratory locust, the total effective temperatures for migratory locust was 1437 DD based on 24-hours integral calculation, so the standard DD was 1271 heat unit when the overflow DD and ineffective DD were taken off, as follow: standard DD = (1437-38-128), and it would take ∼635 DD at least to finish a life cycle for *L. m. manilensis* in this location (Fig. 7).

In addition, we predicted hatchlings emergence mostly by air temperature as we lacked ground temperature data in field, so the DD was 1566 heat units when only based on air temperature (Fig. 7). Thus, it would take ∼700 DD to finish a life cycle for *L. m. manilensis*, as follow: standard DD = (1566-38-128)/2.

### Validation the improved DD model

To further investigate locust voltinism in our growth chamber experiments (Table 3), we recalculated the actual DD in each chamber by the imporved DD model. The results (Table 6) showed that the DD for migratory locust development at Baiquan (BQ), Chengde (CD), Tumotezuoqi (TM), Wenan (WA), Rongan (RA), Qiongzhong (QZ), and Qiongshan (QS) was only 583, 706, 741, 1512, 2040, 2674, and 3351 heat units during locust growing season. They could produce 0.83, 1.01, 1.06, 2.16, 2.91, 3.82, and 4.79 generations, respectively, which were compared to the standard DD (∼700DD). This conclusion matched with the realized voltinism (Table 3), and revealed females require temperature higher than 21°C for reproduction behavior (Table 4).

**Table 6:** DD for migratory locust development at Baiquan (BQ), Chengde (CD), Tumotezuoqi (TM), Wenan (WA), Rongan (RA), Qiongzhong (QZ), and Qiongshan (QS) were recalculated by the improved DD model.

	n	Total DD	Overflow DD	Ineffective DD for egg-laying	Actual DD	Generations completed
BQ	1	698	0	115	583	0.83
CD	1	820	0	114	706	1.01
TM	1	902	0	161	741	1.06
WA	2	1627	7	108	1512	2.16
RA	3	2392	20	332	2040	2.91
QZ	4	3248	5	569	2674	3.82
QS	5	3898	47	500	3351	4.79

The lowercase letter ‘n’ represents the theory generations of *L. m. manilensis* at each location. Actual DD = Total DD-(Overflow DD + Ineffective DD for egg-laying). Generations completed as follow: (n-1)+[actual DD -700*(n-1)]/700DD.

For the voltinism of BQ, it was ∼0.83 as we have deleted invalid DD (Table 6). While it was ∼0.95 by locusts were developing to adult and staying in this stage in a long time without laid eggs (Table 3). Thus, we considered there was no difference between them, because the oriental migratory locusts were unable to breed in BQ, the same as RA, QZ, and QS. For the overflow DD in table 6, they were not revealing the actual values in each location. We have set the 24-hours temperature changes as Fig. 1 of each day as (mean value ±5°C), which were according with normal distribution, however, they were not same as temperature vary trends in China. It is known that temperature different with latitude moves, i.e.for the vary range of 24-hours temperature, high temperature inteval in southern China is more than northern China [56]. Thus, we speculated the overflow DD would be higher than the calculated values in Table 6, especially in southern China. For applying our model, we need to monitor the 24-hours temperature changes of migratory locust at different locations in future.

## Conclusions

In this report, we study various characteristics of the temperature response of the migratory locust including overflow temperature and the effective temperature requirements for egg-laying. Results show that development rate increases with the temperature from 18 to 32°C, temperatures>32°C is overflow temperature for migratory locust development and if not considered as a cut of for heat accumulations may be a cause of overflow in the DD model. Temperature <21°C is not suitable for adults eggs-laying and should be deducted when calculating the DD for adults ovipositing. They are defined as invalid DD which are the key factors that affect the prediction of migratory locust occurrence and result in DD nonlinear increasing with generations arising in field.

Moreover, we propose an integral calculation method to calculate the DD of migratory locust, which also can be used to calculate overflow DD. This metod records 24-h temperature as the basic data to simulate the daily temperature changes and the accuracy is higher than using the daily mean, maximum and minimum temperatures in the data simulation. We also introduce the concept of degree-hours, which improved the accuracy of the DD calculation in areas with temperature variations.

Thirdly, to eliminate the calculating error by traditional DD model, we improve it by studying temperature response of the migratory locust and proposing integral calculation method. Then we constructed a new DD model as: 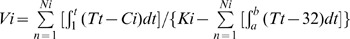. The new model is according with the principle of the traditional DD model, meanwhile it would be more precisely when forecasting the occurrence period of migratory locust.This method can also be used to predict the occurrence period for other pests.
